# Beyond the Cancellation: Identifying Avoidable and Non-avoidable Causes of Elective Operation Theatre Cancellations in a Tertiary Care Hospital

**DOI:** 10.7759/cureus.112889

**Published:** 2026-07-17

**Authors:** Avinash Prakash, Prakash G Gondode, Omshubham G Asai, Rounak Dubey, Nitin Marathe, Ashutosh Kumar, Barkha Agrawal, Anita Yadav

**Affiliations:** 1 Anaesthesiology and Critical Care, All India Institute of Medical Sciences, Nagpur, Maharashtra, IND; 2 Transfusion Medicine, All India Institute of Medical Sciences, Nagpur, Maharashtra, IND; 3 Hospital Administration, All India Institute of Medical Sciences, Nagpur, Maharashtra, IND; 4 Anesthesiology and Critical Care, All India Institute of Medical Sciences, Nagpur, Maharashtra, IND; 5 Obstetrics and Gynaecology, All India Institute of Medical Sciences, Nagpur, Maharashtra, IND

**Keywords:** avoidable cancellation, elective surgery, operating theatre utilization, operation theatre cancellation, pareto analysis, quality improvement

## Abstract

Background

Elective operation theatre (OT) cancellations are an important indicator of perioperative quality and healthcare efficiency. Most studies describe the frequency and causes of cancellations without distinguishing between avoidable and non-avoidable events, limiting the identification of actionable targets for quality improvement. This study aimed to classify elective OT cancellations according to avoidability and identify the major modifiable causes.

Methods

This retrospective observational study was conducted at the All India Institute of Medical Sciences (AIIMS), Nagpur, Maharashtra, India. All elective surgical procedures cancelled after formal OT scheduling between December 2024 and June 2025 were included. Data were obtained from the OT cancellation registry, electronic medical records, anaesthesia records and patient case files. Cancellations were classified as avoidable, non-avoidable or unclassified using predefined criteria. Pareto analysis was performed to identify the principal modifiable causes of avoidable cancellations.

Results

Of 2,744 scheduled elective procedures, 247 were cancelled, resulting in an overall cancellation rate of 9.0%. Among the cancelled cases, 153 (61.9%) were classified as avoidable, 42 (17.0%) as non-avoidable, and 52 (21.1%) as unclassified because of insufficient documentation. Administrative and scheduling-related factors accounted for the largest proportion of cancellations (46.6%). Lack of OT time was the leading avoidable cause (59.5%), followed by inadequate preoperative preparation (9.2%), OT scheduling/admission-related issues (7.2%), and unavailability of intensive care unit beds (5.2%). Pareto analysis showed that these four factors accounted for over 80% of avoidable cancellations.

Conclusion

Most elective OT cancellations were attributable to potentially preventable system-related factors. Targeted interventions focusing on OT scheduling, preoperative assessment, multidisciplinary communication and resource planning may reduce avoidable cancellations, improve OT utilization and enhance perioperative care.

## Introduction

Elective surgery is an integral component of modern healthcare, and the efficient utilisation of operating theatre (OT) resources is essential for delivering timely, safe, and cost-effective surgical care. However, cancellation of scheduled elective surgeries remains a persistent challenge worldwide, with reported cancellation rates ranging from 5% to 40% depending on the healthcare setting, case mix, and institutional efficiency [1,2]. Elective OT cancellation is widely recognised as an important quality indicator because it reflects the effectiveness of perioperative planning, resource allocation, communication and multidisciplinary coordination [3].

The consequences of elective surgery cancellations extend beyond the operating room. For healthcare institutions, cancelled procedures result in underutilization of OT resources, inefficient use of manpower, prolonged waiting lists, and disruption of planned surgical schedules [4]. OTs constitute one of the most resource-intensive areas of a hospital, and cancellations impose a considerable financial burden on healthcare systems, particularly in resource-constrained settings [5].

Patients and their families also experience considerable adverse effects following surgery cancellations. Most patients undergo extensive preoperative preparation and often travel long distances after waiting several weeks or months for an elective procedure. A last-minute cancellation can lead to psychological distress, financial hardship due to repeated travel and loss of wages, delayed treatment, prolonged hospital stay and in some cases, worsening of the underlying disease. Repeated cancellations may further reduce patient confidence in the healthcare system and negatively affect overall patient satisfaction [6].

Previous studies have identified multiple reasons for elective OT cancellations, including patient-related factors, administrative deficiencies, inadequate preoperative assessment, shortage of hospital or intensive care unit beds, equipment failure, staffing constraints and emergency surgical prioritisation [1,5,7,8]. Although these studies have comprehensively described the frequency of various causes, most have reported cancellations as a single heterogeneous outcome without differentiating between avoidable and non-avoidable events [2]. This limits the ability of hospitals to identify which cancellations are amenable to quality improvement interventions.

Distinguishing avoidable from non-avoidable cancellations is essential because not all cancellations represent failures of the healthcare system. While acute deterioration in a patient's clinical condition or emergency surgical priorities are often unavoidable, many cancellations arise from modifiable system-related issues such as incomplete preoperative optimisation, scheduling errors, documentation deficiencies, communication gaps, or inadequate resource planning [2,8]. Identifying these preventable causes enables healthcare organisations to implement targeted interventions using structured quality improvement methodologies such as root cause analysis, Fishbone (Ishikawa) diagrams, Pareto analysis and Plan-Do-Study-Act (PDSA) cycles [9,10]. Such interventions have the potential to improve OT utilisation, reduce unnecessary cancellations, enhance patient satisfaction, and optimise healthcare resource allocation.

Evidence from Indian tertiary care teaching hospitals specifically examining the preventability of OT cancellations remains limited. Therefore, this study aimed to classify elective OT cancellations into avoidable and non-avoidable categories using predefined quality improvement criteria and to identify the major modifiable factors contributing to preventable cancellations. The findings are expected to provide actionable evidence for designing targeted quality improvement strategies to enhance perioperative efficiency and improve the quality of surgical care in tertiary healthcare settings.

## Materials and methods

This retrospective observational study was conducted at the Department of Anaesthesiology and Critical Care, All India Institute of Medical Sciences (AIIMS), Nagpur, Maharashtra, India. The study evaluated elective OT cancellations across the Central Operation Theatre Complex of the institute.

**Figure 1:**
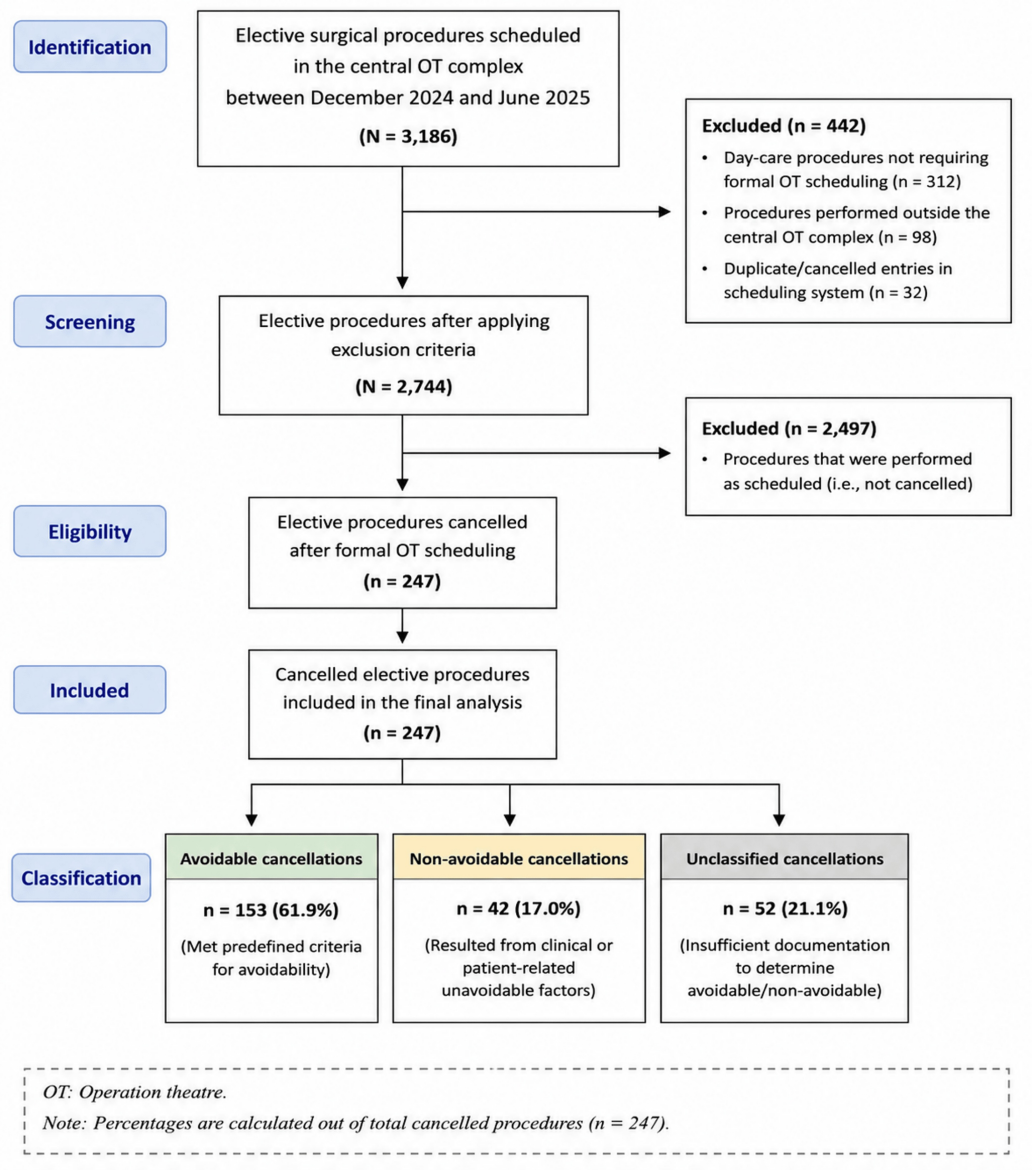
Strengthening the Reporting of Observational studies in Epidemiology (STROBE) flow diagram of patient selection and classification of elective operation theatre cancellations.

The study included all patients of any age or sex whose elective surgical procedures were cancelled after formal OT scheduling between December 2024 and June 2025. The study population comprised all elective surgical procedures scheduled through the Central Operation Theatre Complex (first-floor OT complex) of the institute during the study period. Elective procedures from General Surgery, Orthopaedics, Neurosurgery, Urology, Plastic Surgery, Paediatric Surgery, Surgical Oncology, Gastrointestinal Surgery, Obstetrics and Gynaecology, Otorhinolaryngology, and other surgical specialities were included. Emergency surgeries, day-care procedures not requiring formal OT scheduling, and procedures performed outside the Central Operation Theatre Complex were not included in the study population. The process of patient selection and inclusion in the study is illustrated in Figure 1.

As this was a retrospective observational study, all consecutive eligible elective OT cancellations during the study period were included in the analysis. Therefore, a formal sample size calculation was not applicable. A total of 247 elective procedures were cancelled among 2,744 scheduled elective surgeries.

Data were retrieved from the institutional Hospital Management Information System (HMIS), which serves as the electronic medical record system, along with the OT cancellation registry, anaesthesia records, and patient case files using a structured case record form. Information collected included patient demographics (age and sex), surgical speciality, scheduled procedure, stage of cancellation, person initiating cancellation, documented reason for cancellation, rescheduling status, and patient outcome where available. All records were independently reviewed by the investigators, and discrepancies were resolved by consensus after verification with the original hospital records.

The primary outcome was the classification of cancellations as avoidable or non-avoidable using predefined criteria developed from published literature and multidisciplinary consensus involving anaesthesiologists, surgeons, and OT nursing personnel.

The avoidability of each cancellation was independently assessed by two investigators using the predefined criteria. Cases in which the principal cause of cancellation was unclear or where disagreement existed between the two investigators were reviewed by an independent senior consultant anaesthesiologist, who was not a member of the study team and was not involved in the initial data collection or classification. The final classification was assigned after adjudication using the predefined criteria and verification against the original hospital records. The classification of cancellations as avoidable or non-avoidable was based on predefined criteria adapted from previously published literature on elective surgical cancellations and quality improvement in perioperative care [6,8]. Avoidable cancellations were defined as those resulting from potentially modifiable system- or process-related factors, including incomplete preoperative evaluation, inadequate patient optimisation, incomplete documentation, scheduling errors, lack of informed consent, equipment or instrument unavailability, OT scheduling conflicts, staffing shortages and communication or resource-planning deficiencies. Inter-rater agreement statistics were not prospectively recorded. Therefore, measures, such as Cohen's kappa, could not be calculated retrospectively.

Non-avoidable cancellations were defined as those resulting from unforeseen clinical circumstances despite appropriate perioperative planning, such as sudden deterioration in the patient's medical condition, newly detected illness precluding surgery, emergency case prioritisation, or acute infectious illness. Where multiple reasons were documented, the principal cause was assigned after multidisciplinary review involving the study investigators, the treating surgeon and an independent senior consultant anaesthesiologist, using the original hospital records and predefined classification criteria. Cancellation causes were subsequently grouped into five broad domains: administrative/scheduling, patient/preoperative preparation, clinical/patient condition, emergency priority, and equipment/resource-related factors. Pareto analysis was performed to identify the relatively small number of modifiable causes responsible for the majority of avoidable cancellations.

The primary outcome was the proportion of elective OT cancellations classified as avoidable and non-avoidable. Secondary outcomes included the distribution of cancellation causes, stage of cancellation, personnel responsible for cancellation decisions, rescheduling status and identification of major avoidable causes requiring quality improvement interventions.

Data were entered and analysed using Microsoft Excel (Microsoft Corporation, Redmond, WA, USA). Categorical variables were summarised as frequencies and percentages. Pareto analysis was performed to rank avoidable causes of elective OT cancellations according to their frequency and cumulative percentage, thereby identifying the principal modifiable contributors for prioritising quality improvement interventions. The study was approved by the Institutional Ethics Committee of AIIMS Nagpur (approval no. IEC/Pharmac/2025/1393, dated July 2, 2025). Patient confidentiality was maintained by anonymising all extracted data before analysis.

## Results

During the study period, a total of 2,744 elective surgical procedures were scheduled, of which 247 (9.0%) were cancelled, resulting in an overall elective OT cancellation rate of 9.0%. These 247 cancelled procedures were included in the final analysis. The baseline demographic and clinical characteristics of the study population are summarised in Table 1. The highest proportion of cancellations occurred among patients aged 36-45 years (44; 17.8%), followed by those aged one to 10 years (40; 16.2%) and 46-55 years (37; 15.0%). Male patients constituted 140 (56.7%) of the study population, while females accounted for 107 (43.3%).

**Table 1:** Baseline demographic and clinical characteristics of patients with elective operation theatre cancellations

Variable	Number (n)	Percentage (%)
Age group (years)		
<1	8	3.2
1-10	40	16.2
11-18	34	13.8
19-25	14	5.7
26-35	23	9.3
36-45	44	17.8
46-55	37	15.0
56-65	31	12.6
>65	16	6.5
Total	247	100.0
Sex		
Male	140	56.7
Female	107	43.3
Decision-maker for cancellation		
Surgeon	141	57.1
Anaesthesiologist	64	25.9
Surgeon and anaesthesiologist	32	13.0
Patient/family	4	1.6
Not specified	6	2.4
Rescheduled surgery		
Yes	198	80.2
No	15	6.1
Uncertain	34	13.8
Avoidability classification		
Avoidable	153	61.9
Non-avoidable	42	17.0
Unclassified	52	21.1

The decision to cancel surgery was most commonly made by the surgeon (141; 57.1%), followed by the anaesthesiologist (64; 25.9%), whereas joint decisions by both the surgeon and anaesthesiologist accounted for 32 (13.0%) cancellations. Following cancellation, 198 (80.2%) patients were subsequently rescheduled for surgery, while 15 (6.1%) were not rescheduled, and 34 (13.8%) had an uncertain rescheduling status. Based on the predefined quality improvement criteria, 153 (61.9%) cancellations were classified as avoidable, 42 (17.0%) as non-avoidable, and 52 (21.1%) remained unclassified because of insufficient documentation (Table 1).

The distribution of cancellation causes according to broad domains is presented in Table 2. Administrative and scheduling-related issues constituted the largest category, accounting for 115 (46.6%) cancellations. Patient-related factors, including inadequate preoperative preparation, contributed to 26 (10.5%) cancellations, while 25 (10.1%) cancellations were attributed to changes in the patient's clinical condition. Emergency surgical prioritisation accounted for 17 (6.9%) cases, whereas equipment or resource-related factors were responsible for 12 (4.9%) cancellations. The remaining 52 (21.1%) cases could not be categorised because of incomplete documentation.

**Table 2:** Distribution of elective operation theatre cancellations according to broad cause domains

Cause domain	Number ( n)	Percentage (%)
Administrative / scheduling	115	46.6
Patient / preoperative preparation	26	10.5
Clinical / patient condition	25	10.1
Emergency priority	17	6.9
Equipment / resource-related	12	4.9
Unclassified	52	21.1

**Figure 2:**
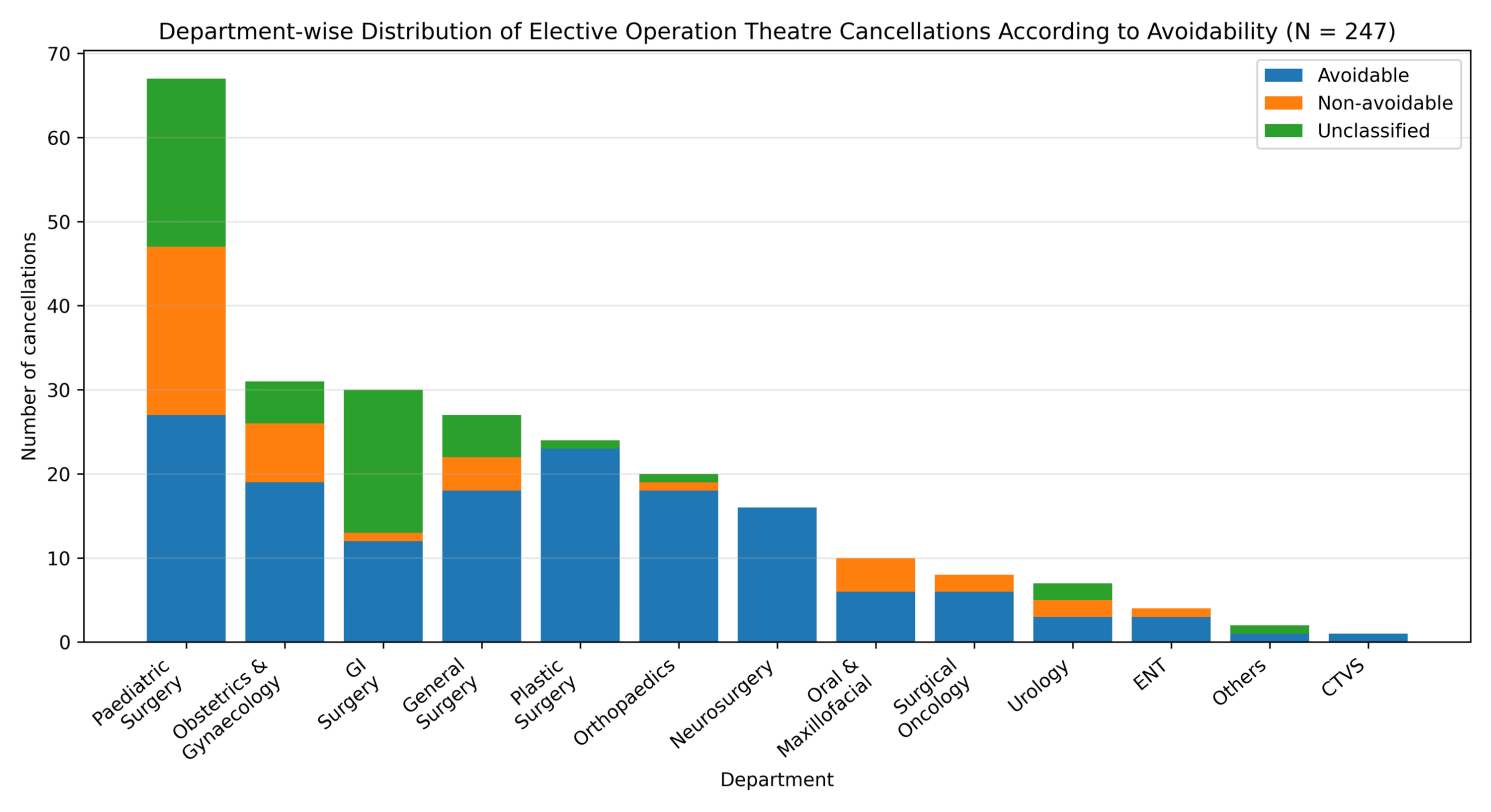
Department-wise distribution of avoidable and non-avoidable elective operation theatre cancellations.

Department-wise analysis demonstrated variability in the proportion of avoidable and non-avoidable cancellations across surgical specialties, with higher-volume departments contributing the majority of avoidable cancellations (Figure 2). However, because department-wise denominator data (total scheduled elective procedures per specialty) were unavailable, specialty-specific cancellation rates could not be calculated.

Among the avoidable cancellations, lack of OT time emerged as the predominant cause, accounting for 91 (59.5%) cases (Table 3). Other important preventable causes included inadequate preoperative preparation (14; 9.2%), OT scheduling or admission-related issues (11; 7.2%), unavailability of ICU beds (8; 5.2%), incomplete documentation (5; 3.3%), and scheduling errors (5; 3.3%). Less frequent causes included lack of informed consent, patient refusal or non-compliance, and equipment failure.

**Table 3:** Frequency and proportion of major avoidable causes of elective operation theatre cancellation.

Avoidable cause	Number ( n)	Percentage (%)
Lack of OT time	91	59.5
Inadequate preoperative preparation	14	9.2
OT time/scheduling / admission-related issue	11	7.2
Unavailability of ICU beds	8	5.2
Documentation incomplete	5	3.3
Scheduling error	5	3.3
Lack of consent	3	2.0
Patient refusal / non-compliance	2	1.3
Equipment failure	2	1.3
Other avoidable causes	12	7.8
Total	153	100

**Figure 3:**
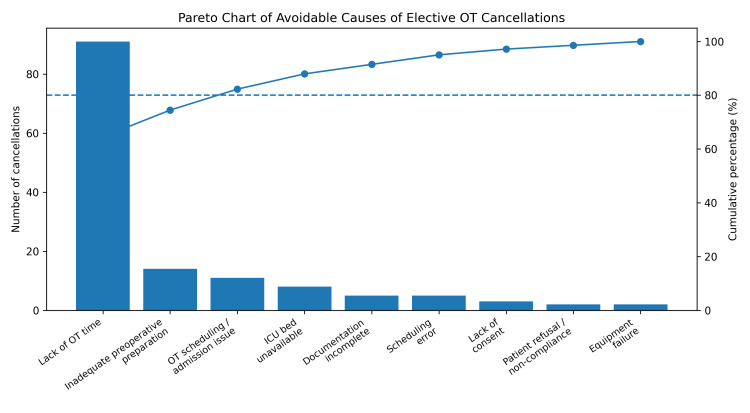
Pareto analysis of avoidable causes of elective operation theatre cancellations demonstrating the cumulative contribution of major modifiable factors.

Pareto analysis demonstrated that a limited number of modifiable causes accounted for the majority of avoidable cancellations. Specifically, lack of OT time, inadequate preoperative preparation, scheduling or admission-related issues, and ICU bed unavailability together contributed to more than 80% of all avoidable cancellations, highlighting these factors as priority targets for quality improvement interventions (Figure 3).

The association between the stage of cancellation and avoidability is shown in Table 4. Most cancellations occurred on the day of surgery before the patient entered the OT (215/247; 87.0%), with 138 of these classified as avoidable. By contrast, cancellations occurring after patient transfer to the OT were relatively uncommon. Among the 12 cancellations occurring before induction of anaesthesia inside the OT, equal numbers were classified as avoidable and non-avoidable. Only one cancellation occurred after induction of anaesthesia, and this was classified as non-avoidable. These findings indicate that the majority of preventable cancellations were identified late in the perioperative pathway, after substantial hospital resources had already been committed.

**Table 4:** Distribution of elective operation theatre (OT) cancellations according to stage of cancellation and avoidability classification

Stage of cancellation	Avoidable	Non-avoidable	Unclassified	Total
Pre-operative before admission	6	2	4	12
On day of surgery before entering OT	138	32	45	215
In OT before anaesthesia	6	6	0	12
In OT after anaesthesia	0	1	0	1
Other	3	1	3	7

Overall, the results indicate that a substantial proportion of elective OT cancellations were attributable to modifiable organisational and administrative factors rather than unavoidable clinical circumstances. The predominance of avoidable cancellations and the concentration of these events around a few key causes suggest considerable scope for targeted quality improvement initiatives aimed at optimising OT scheduling, strengthening preoperative assessment, improving documentation, and enhancing perioperative resource planning.

## Discussion

The overall elective OT cancellation rate in the present study was 9.0%, which lies within the range reported from tertiary care hospitals worldwide and is comparable with findings from several recent studies. Abate et al., in their systematic review and meta-analysis, reported that elective surgery cancellation rates vary widely across healthcare settings, ranging from approximately 5% to over 30%, reflecting differences in hospital infrastructure, perioperative processes, resource availability, and healthcare systems [1]. Similarly, Koushan et al. highlighted substantial international variation in cancellation rates and identified organisational factors, including OT scheduling, resource allocation, and perioperative planning, as major determinants of cancellation rates across institutions [2]. The observed cancellation rate in our study suggests reasonably efficient OT utilisation; however, the high proportion of avoidable cancellations indicates considerable scope for improving perioperative processes through targeted quality improvement initiatives.

The present study evaluated elective OT cancellations using a quality improvement framework by classifying cancellations as avoidable and non-avoidable to identify modifiable factors amenable to targeted interventions. The principal finding was that 153 (61.9%) of the 247 cancelled procedures were classified as avoidable, and among the 195 classifiable cancellations (excluding 52 unclassified cases), 153 (78.5%) were considered potentially preventable.

Administrative and scheduling deficiencies constituted the largest category of cancellations, with lack of OT time emerging as the single most common avoidable cause. Furthermore, the Pareto analysis demonstrated that four modifiable factors - lack of OT time, inadequate preoperative preparation, OT scheduling/admission-related issues, and ICU bed unavailability - accounted for more than 80% of avoidable cancellations. These findings indicate that relatively focused interventions could substantially improve OT efficiency and reduce preventable cancellations in tertiary care hospitals [1,2,10,11].

Elective surgery cancellation remains a global challenge despite advances in perioperative care. A recent systematic review by Koushan et al. concluded that cancellation rates are influenced by a combination of patient-related, organisational, and resource-related factors, with administrative inefficiencies representing one of the most important contributors [2]. The authors also emphasised that distinguishing unavoidable from avoidable cancellations provides a more meaningful basis for quality improvement than reporting crude cancellation rates alone. Our findings support this concept by demonstrating that a substantial proportion of cancellations were potentially preventable and therefore amenable to targeted interventions.

Our results are also consistent with the systematic review and meta-analysis by Abate et al., which reported that the majority of same-day elective surgery cancellations worldwide were attributable to preventable causes, particularly inadequate preoperative assessment, scheduling problems and organisational deficiencies [1]. The authors suggested that many of these cancellations could be avoided through better perioperative planning, structured preoperative assessment clinics and improved communication among multidisciplinary teams. The predominance of avoidable cancellations in our study reinforces these observations and highlights the continued need for systematic quality improvement initiatives in tertiary care hospitals.

An important finding of the present study was that 52 (21.1%) cancellations remained unclassified because the available documentation did not provide sufficient information to identify the principal reason for cancellation. It is possible that some of these cases represented potentially avoidable cancellations; however, the available records did not permit reliable classification without introducing the risk of misclassification. Therefore, these cases were conservatively retained as unclassified. This highlights an important deficiency in routine OT documentation and emphasises the need for standardised recording of cancellation reasons. Improving the quality and completeness of documentation would not only facilitate more accurate audits but also enable better identification of system-level deficiencies and monitoring of quality improvement interventions over time.

One of the most important findings of the present study was that administrative and scheduling-related factors, considered collectively as a broad category of avoidable causes, accounted for the largest share of cancellations. Within this category, lack of OT time was the most common individual cause, accounting for 91 (59.5%) of the 153 avoidable cancellations. Similar findings have been reported by Cho et al., who identified scheduling inefficiencies, prolonged operating lists and inadequate operating room time as major contributors to elective surgery cancellations during a decade-long hospital audit [5]. Likewise, Ogwal et al. demonstrated that facility-related factors were responsible for approximately two-thirds of elective surgery cancellations, with insufficient OT time representing the commonest cause [12]. These findings consistently suggest that realistic scheduling, accurate estimation of operative duration, and effective coordination between surgical and anaesthesia teams are fundamental to improving OT efficiency.

The present study also identified inadequate preoperative preparation as the second most frequent avoidable cause of cancellation. This observation is supported by McKendrick et al., who demonstrated that the introduction of structured preoperative preparation clinics significantly reduced same-day cancellations, particularly those related to medical optimisation and failure to attend surgery [9]. More recently, Pattnaik et al. reported that implementation of a quality improvement programme incorporating pre-anaesthesia assessment clinics, individualised anaesthetic planning, preoperative checklists and repeated patient evaluation successfully reduced cancellation rates in an Indian tertiary care hospital [13]. Collectively, these findings support strengthening pre-anaesthesia assessment services, standardizing preoperative investigations, and ensuring timely optimization of comorbidities to minimize preventable cancellations.

Another important observation was that 215 (87.0%) of the 247 elective OT cancellations occurred on the scheduled day of surgery before patient transfer to the OT, and the majority of these (138/215, 64.2%) were classified as avoidable. This indicates that deficiencies were identified relatively late in the perioperative pathway, after patients had completed admission procedures and hospital resources had already been committed. Similar observations have been reported by Kaddoum et al., whose prospective audit demonstrated that nearly three-quarters of day-of-surgery cancellations were potentially avoidable and predominantly resulted from hospital-related factors such as incomplete medical evaluation and operating room delays [14]. Likewise, Tan et al. reported that late cancellations were strongly associated with deficiencies in preoperative assessment and emphasised the protective role of formal anaesthesia evaluation before surgery [10]. These findings highlight the importance of early verification of documentation, patient readiness, and preoperative checklists to detect modifiable problems before the day of surgery.

The predominance of day-of-surgery cancellations suggests that several modifiable workflow issues may have remained undetected until the final preoperative stage. Although the present study did not capture detailed process indicators such as the timing of pre-anaesthesia assessment, admission logistics or preoperative checklist compliance, these factors may have contributed to the late identification of administrative, scheduling and patient-related issues. Future prospective studies incorporating detailed workflow mapping and perioperative process indicators are needed to better understand these factors and inform targeted quality improvement interventions.

The distribution of avoidable cancellations varied across surgical specialties. However, because department-wise denominators (total scheduled elective procedures per specialty) were unavailable, these findings represent the distribution of cancelled cases rather than specialty-specific cancellation rates. Therefore, comparisons between specialties should be interpreted with caution. Departments with larger operative volumes demonstrated a greater burden of preventable cancellations, primarily related to scheduling constraints and OT availability. Similar specialty-specific variation has been described by Tayeb, who reported that the profile of cancellations differed considerably among surgical specialties and emphasised the need for periodic institutional audits to identify local causes and guide targeted interventions [3]. These findings support tailoring quality improvement initiatives according to specialty-specific workflow rather than adopting a uniform hospital-wide strategy.

A notable strength of the present study is the application of Pareto analysis to prioritise quality improvement efforts. Rather than simply listing causes of cancellation, Pareto analysis identified a limited number of modifiable factors responsible for the majority of avoidable events. This approach provides practical guidance for hospital administrators by enabling focused interventions on high-impact areas. Similar quality improvement strategies have been advocated by Antoniou et al., who demonstrated improved theatre utilisation after implementing targeted scheduling interventions, and by Pattnaik et al., who successfully employed Plan-Do-Study-Act (PDSA) cycles, root cause analysis, and structured preoperative assessment to reduce surgical cancellations. These findings support the use of continuous audit, root cause analysis, and Pareto-based prioritisation to enhance OT efficiency [4,13].

From the patient perspective, the high proportion of patients requiring rescheduling after cancellation highlights the substantial clinical and psychological burden associated with delayed surgery. Repeated hospital visits, prolonged waiting periods, uncertainty regarding treatment, and delayed surgery adversely affect patient satisfaction and may contribute to disease progression in selected cases. Both Abate et al. and Koushan et al. emphasised that surgery cancellations adversely affect not only hospital efficiency but also patient well-being, resulting in emotional distress, disruption of daily activities, and reduced confidence in healthcare services [1,2].

Our findings are also consistent with reports from the Indian healthcare setting. Kumar and Gandhi reported that administrative deficiencies, inadequate preoperative preparation and scheduling-related issues were the predominant causes of same-day elective surgery cancellations in a multidisciplinary tertiary care hospital, highlighting the need for better perioperative planning and coordination [15]. Similarly, Pattnaik et al. demonstrated that structured preoperative assessment and quality improvement interventions significantly reduced preventable surgical cancellations in an Indian tertiary care centre [13]. Collectively, these studies reinforce the applicability of our findings to the Indian healthcare system and support targeted quality improvement initiatives to enhance OT efficiency and patient care.

The findings of the present study provide several practical opportunities for quality improvement. Administrative and scheduling deficiencies can be addressed through optimised OT scheduling, realistic case-time estimation, and periodic review of operating lists. Inadequate preoperative preparation may be reduced by strengthening pre-anaesthesia assessment clinics and standardised preoperative checklists. Documentation-related cancellations can be minimised through electronic verification and mandatory completion of perioperative documentation before OT scheduling. Improved multidisciplinary communication, proactive ICU bed allocation and resource planning for equipment and staffing may further reduce preventable cancellations. As demonstrated by the Pareto analysis, prioritising these high-frequency modifiable causes is likely to achieve the greatest reduction in avoidable cancellations.

The present study has several strengths. First, it is among the few Indian studies to classify elective OT cancellations into avoidable and non-avoidable categories using predefined quality improvement criteria rather than reporting only descriptive frequencies. Second, the inclusion of multiple surgical specialities provides a comprehensive overview of cancellation patterns in a large tertiary care teaching hospital. Third, the application of a quality improvement framework incorporating broad cause domains and Pareto analysis enhances the practical relevance of the findings by identifying priority areas for intervention rather than merely describing the problem. Finally, the use of standardised data extraction and multidisciplinary consensus for classification improved the consistency of cause attribution.

The study should also be interpreted in light of certain limitations. First, it was conducted at a single tertiary care centre, which may limit the generalizability of the findings to hospitals with different patient populations, infrastructure, or scheduling practices. Second, the retrospective design relied on the accuracy and completeness of routine hospital documentation, resulting in 52 (21.1%) cases remaining unclassified because of insufficient information. Consequently, some potentially avoidable cancellations may have remained unclassified, although they were conservatively retained in this category to minimise the risk of misclassification. Third, although avoidability was assessed independently using predefined criteria and consensus, formal inter-rater reliability statistics were not prospectively recorded, and the classification inevitably involved some degree of subjective judgement. Fourth, department-wise analyses were based on the distribution of cancelled procedures because specialty-specific data on the total number of scheduled elective surgeries were unavailable; therefore, true specialty-specific cancellation rates could not be determined. Additionally, the study primarily employed descriptive statistical analyses, including Pareto analysis and did not perform inferential analyses such as chi-square tests or multivariable logistic regression to identify independent predictors of avoidable cancellations. Therefore, the findings should be interpreted primarily as descriptive observations intended to guide quality improvement initiatives. Finally, the study did not evaluate the effectiveness of specific quality improvement interventions or measure changes in cancellation rates following implementation of corrective strategies. Prospective multicentre studies incorporating standardised avoidability criteria, formal assessment of inter-rater agreement, and evaluation of post-intervention outcomes are warranted to validate these findings and determine the effectiveness of targeted quality improvement programmes.

## Conclusions

This study demonstrated that a substantial proportion of elective OT cancellations in a tertiary care teaching hospital were classified as avoidable, with administrative and scheduling deficiencies accounting for the largest share of preventable cancellations. Pareto analysis identified a small number of modifiable system-level factors responsible for most avoidable cancellations, highlighting priority areas for quality improvement. These findings provide a practical framework for prioritising targeted quality improvement initiatives to reduce preventable elective surgery cancellations. Future prospective multicentre studies using standardised avoidability criteria and evaluating the impact of quality improvement interventions are warranted.
